# Population pharmacokinetics of cabazitaxel in patients with advanced solid tumors

**DOI:** 10.1007/s00280-012-2058-9

**Published:** 2013-01-09

**Authors:** Géraldine M. Ferron, Yang Dai, Dorothée Semiond

**Affiliations:** 1 GlaxoSmithKline, 709 Swedeland Road, King of Prussia, PA 19406 USA; 2 Vertex Pharmaceuticals, 130 Waverly Street, Cambridge, MA 02139 USA; 3 Sanofi, 13 quai Jules Guesde, BP14, 94403 Vitry-sur-Seine Cedex, France

**Keywords:** Cabazitaxel, Chemotherapy, Taxane, Population pharmacokinetics, Advanced solid tumors, Urogenital cancer

## Abstract

**Purpose:**

To develop a population pharmacokinetic (PK) model for cabazitaxel in patients with advanced solid tumors and examine the influence of demographic and baseline parameters.

**Methods:**

One hundred and seventy patients who received cabazitaxel (10–30 mg/m^2^, 1-h IV infusion) every 7 or 21 days in five Phase I–III studies were analyzed by non-linear mixed-effect modeling (NONMEM VI). Model evaluation comprised non-parametric bootstrap and visual predictive checks.

**Results:**

Cabazitaxel PK was best described by a linear three-compartment model with: first-order elimination; interindividual variability on clearance (CL), central volume of distribution (V1), and all intercompartmental rate constants except K21; interoccasion variability in CL and V1; proportional residual error of 27.8 %. Cabazitaxel CL was related to body surface area (BSA) and tumor type (breast cancer; finding confounded by study). Typical CL for a non-breast cancer patient with a BSA of 1.84 m^2^ was 48.5 L/h, with V1 26.0 L, steady-state volume of distribution 4,870 L and alpha, beta, and gamma half-lives of 4.4 min, 1.6, and 95 h, respectively. Sex, height, weight, age, Caucasian race, renal/hepatic function, and cytochrome P450 inducer use did not significantly further explain the PK of cabazitaxel. Bootstrap and posterior predictive checks confirmed the adequacy of the model.

**Conclusions:**

Cabazitaxel PK appears unaffected by most baseline patient factors, and the influence of BSA on CL is addressed in practice by BSA-dependent doses. This analysis suggests consistent cabazitaxel PK and exposure across most solid tumor types, although the potential influence of breast cancer on CL requires further confirmation.

**Electronic supplementary material:**

The online version of this article (doi:10.1007/s00280-012-2058-9) contains supplementary material, which is available to authorized users.

## Introduction

Cabazitaxel is a novel, semi-synthetic taxane drug that promotes the assembly of tubulin and stabilizes microtubules [1]. This agent has been demonstrated to have comparable potency to docetaxel in a number of murine and human cell lines [2]. Furthermore, cabazitaxel possesses greater potency than docetaxel and paclitaxel in cancer cell lines expressing a multidrug-resistant phenotype [2]. Cabazitaxel has also shown promising in vivo activity in dose–response studies in a wide variety of subcutaneous tumor xenograft models in mice [3].

Cabazitaxel in combination with prednisone/prednisolone is approved in the US, Europe, and Canada for the treatment of men with metastatic hormone-refractory prostate cancer previously treated with a docetaxel-containing regimen. This approval was based on the results of the Phase III TROPIC trial (Treatment of Hormone-Refractory Metastatic Prostate Cancer Previously Treated With a Taxotere-Containing Regimen), in which cabazitaxel in combination with prednisone demonstrated a significant benefit in overall survival compared with mitoxantrone plus prednisone (hazard ratio 0.70; *p* < 0.0001) (clinicaltrials.gov ID: NCT00417079) [4].

Cabazitaxel has a predictable pharmacokinetic (PK) profile similar to that of docetaxel, with dose-proportional exposure and triphasic elimination [1, 5]. In two Phase I studies in which it was administered as a 1-h intravenous (IV) infusion every 3 weeks in patients with advanced solid tumors [1, 5], cabazitaxel exposure showed dose proportionality across the dosing range of 10–30 mg/m^2^. The concentration–time profile was triphasic, with a prolonged terminal phase (mean gamma half-life [*t*_1/2_] 62–77 h). The mean volume of distribution at steady state (*V*_ss_) was very large (mean 2,034–2,484 L/m^2^). Clearance (CL) rates were also high (27.3–44.7 L/h/m^2^). Results from one study suggested that CL is correlated with body surface area (BSA): The observed between-patient variability in CL was slightly reduced when adjusted to BSA [1]. There were some notable differences compared with the PK profile of docetaxel. Specifically, the terminal phase *t*_1/2_ was longer than that of docetaxel (*t*_1/2_ = 11.1 h) and the V_ss_ was substantially greater than that of docetaxel (*V*_ss_ = 61.4 L/m^2^ with an assumed BSA of 1.84 m^2^). CL was also somewhat higher than that of docetaxel (CL = 21 L/h/m^2^) [6]. However, these differences may be due in part to the lower limit of quantitation of cabazitaxel [1].

Cabazitaxel is highly protein bound both in vitro and ex vivo (89–92 %) [5, 7], being mainly bound to human serum albumin (82 %) and lipoproteins (88 % for high-density lipoprotein, 70 % for low-density lipoprotein, and 56 % for very low-density lipoprotein) [7]. It is principally metabolized in the liver through the cytochrome P450 (CYP) 3A4/5 isoenzyme, and seven metabolites have been detected in plasma, each of which accounts for ≤5 % of cabazitaxel exposure and three of which are active [7]. Cabazitaxel is mainly excreted in the feces as numerous metabolites (76 % of the dose) [8].

While early Phase I PK studies are informative, such studies are limited both in the range of covariates they can address and, given the small sample size, in the representativeness of their results to the overall patient population. Population PK approaches that employ sophisticated statistical techniques such as non-linear mixed-effects modeling (as implemented in NONMEM^®^) are particularly useful for examining PK variability. In addition, this approach is applicable to later-stage studies where PK sampling is limited. Modeling approaches are of increasing importance to the drug development process [9], particularly when applied to a broad cross-study patient population. Population PK and PK/pharmacodynamics (PD) evaluations were integrated throughout the clinical development of cabazitaxel [5, 10]; the population PK of cabazitaxel in patients from Phase I to III studies with various advanced solid tumors is described here.

## Methods

### Study design

Data from three Phase I studies in patients with various advanced solid tumors [1, 5, 11], one Phase II study in patients with taxane-resistant breast cancer [10], and one Phase III study in patients with metastatic castration-resistant prostate cancer (mCRPC) refractory to docetaxel [4] were combined for this analysis. Cabazitaxel was administered as a 1-h IV infusion either every 3 weeks or once weekly for the first 4 weeks of a 5-week treatment cycle. Details of the study designs are presented in Tables 1 and 2.

**Table 1:** Studies included in PK modeling

Study number	Phase	Patient type	Number of patients providing samples for PK (total and by cycle)	Administration schedule	Doses	Sampling scheme
TED6188 [5]	I	Advanced solid tumors	Total: 21 Cycle 1: 21 Cycle 2: 20 Cycle 3: 3	Every 3 weeks	10–30 mg/m^2^	Cycles 1 and 2; just before infusion, 30 min after start of infusion, and 5 min before the end of infusion; 5, 15, 30, 60 min, and 2, 4, 6, 10, 24, 48, 72, and 120 h post-infusion
TED6189 [11]	I	Advanced solid tumors	Total: 13^a^ Cycle 1:13	Once weekly for the first 4 weeks of each 5-week treatment cycle	10–12 mg/m^2^	Cycles 1 and 2 on Day 1 and Day 22 (only cycle 1 Day 1 was used); just before infusion, 30 min after start of infusion, and 5 min before the end of infusion; 5, 10, 20, 30, 60, 90 min, and 2, 3, 4, 6, 10, 24, and 48 h post-infusion
TED6190 [1]^b^	I	Advanced solid tumors	Total: 35^c^ Cycle 1: 25 Cycle 2: 33 Cycle 3: 3	Every 3 weeks	10–25 mg/m^2^	Cycles 1, 2, and 3 on Day 1; just before infusion, 30 min after start of infusion, and 5 min before the end of infusion; 5, 15, 30, and 60 min, and 2, 4, 6, 10, 24, 48, 72, 120, 168, and 240 h post-infusion
ARD6191 [10]	II	Taxane-resistant breast cancer (*n* = 84; 34 included in PK analysis)	Total: 34 Cycle 1: 34	Every 3 weeks (*n* = 23) or once weekly for the first 4 weeks of each 5-week treatment cycle (*n* = 11)	10–20 mg/m^2^ increasing to 25 mg/m^2^ after cycle 1 if no Grade > 2 toxicity in cycle 1	Cycle 1 on Day 1; schedule as shown in Table 2
EFC6193 (TROPIC; NCT 00417079) [4]	III	Metastatic castration-resistant prostate cancer refractory to docetaxel (cabazitaxel arm: *n* = 378; 67 included in PK analysis)	Total: 67 Cycle 1: 67 Cycle 2: 51	Every 3 weeks	25 mg/m^2^ (in combination with prednisone 10 mg/day)	Cycles 1 and 2; schedule as shown in Table 2

^a^Total number of patients included in study was greater, but only those receiving a dose of 10–12 mg/m^2^ were included in this analysis

^b^Single-center study

^c^Of these patients, 10 received both oral (cycle 1) and IV administrations (subsequent cycles) (Sanofi. Data on file); only data from cycle 2 (IV administration) were included in the analysis for these patients

**Table 2:** Blood sampling schedule for ARD6191 and EFC6193 studies

Sampling schedule	Sampling time					
	T0: before the infusion	T1: during infusion	Post-infusion			
			T2 (min)	T3 (h)	T4 (h)	T5^a^ (h)
1	Before the infusion	30 min before the end of infusion	5	1	6–10	24–48
2			10	2	8–12	48–72
3		10 min before the end of infusion	20	3	10–14	72–120
4			30	4	12–16	120–168

^a^For EFC6193, after a protocol amendment, the start of the window was 24 h for all sampling schedules

### Sample collection and bioanalytical methods

In the Phase I studies, blood samples for plasma were taken from the patients (*n* = 69) at cycles 1 and 2 according to the schedule shown in Table 1. Where possible, samples were also taken in cycle 3, particularly in cases of intrapatient dose escalation. In the Phase II and III studies, plasma for PK analysis was sampled from a subgroup of patients (Phase II: *n* = 34; Phase III: *n* = 67) over 1–2 cycles. Following the methodology previously described for docetaxel [12], patients were randomly assigned to one of four pre-defined sampling schedules as shown in Table 2. Actual sampling times and actual start and end times of infusions were recorded. For this analysis, only samples taken on day 1 of cycle 1 were used in trials featuring weekly administration of cabazitaxel (TED6189 and ARD6191).

Blood samples were collected in heparinized tubes (lithium heparinate). The samples were centrifuged within 30 min at approximately 2,000 g for 15 min. The plasma was then removed, placed into polypropylene tubes, labeled, frozen, and stored at −20 °C until analysis.

In the Phase I and II studies, cabazitaxel plasma concentrations were analyzed by a validated method comprising an automated solid-phase extraction using a taxane analog as an internal standard followed by liquid chromatography with tandem mass spectrometry detection. The assay accuracy, defined as the percentage difference between the nominal and the mean measured concentrations of quality controls, ranged from −4.1 to 6.4 % in the three Phase I studies [1, 5] (Fumoleau et al. Unpublished results) and ranged from 0 to 7.0 % in the Phase II study (Sanofi. Data on file) over the analysis period. The precision of the assay, established by the coefficients of variation of the quality controls, ranged from 9.5 to 14.0 % in the three Phase I studies [1, 5] (Fumoleau et al. Unpublished results) and from 4.1 to 9.4 % in the Phase II study (Sanofi. Data on file) over the analysis period. In the Phase III trial, the method was slightly adapted (the mass spectrometer instrument was upgraded and the taxane analog was replaced by [^2^H_6_]-cabazitaxel as the internal standard). The accuracy was in the range −3.25 to 0 % and the precision ranged from −3.81 to 4.19 % over the analysis period. The lower limit of quantification (LOQ) for both methods was 1.0 ng/mL. Concentrations below the lower LOQ were not included in this analysis.

### Population PK analysis

#### Software

The population PK analysis was performed using a non-linear mixed-effect model approach implemented in the NONMEM^**®**^ computer program (version VI, level 1.2, ICON, Hanover, MD, USA) [13] running on a Linux cluster [14]. All runs were performed using the first-order conditional estimation method with the interaction option selected. Graphical and all other statistical analyses were performed using *R* (≥2.10).

#### Outlier detection

The identification of potential outliers in the initial dataset was performed using conditional-weighted residuals and individual-weighted residuals obtained from a preliminary three-compartment model. The box plot examination and the T-procedure (also named Grubb’s test, *p* < 0.05) were used [15]. Once outlier concentrations were excluded, the initial dataset became the total dataset. The final full model was rerun with the initial dataset.

#### Structural model

Based on previous modeling performed on Phase I studies [1, 5] and exploratory graphical analysis, two- and three-compartment structural kinetic models with first-order elimination were planned for evaluation. Parameterization in terms of CLs and volumes, or volumes and rate constants, were also compared. The best structural model was chosen on the basis of the examination of objective function and the visual inspection of standard goodness-of-fit plots, including the individual fits.

#### Statistical model

As most patients had PK sampling on more than one occasion, interoccasion variability (IOV) was evaluated for CL and V1. A cycle was defined as an occasion. Interindividual variability (IIV) and IOV in a PK parameter, P, were included in the model and assumed to be log-normally distributed, according to Eq. (1):

1 $$ P_{jk} = {\text{TVP}} \cdot e^{(\eta j + \tau k)} $$

where *P*_*jk*_ is an individual PK parameter for the *j*th individual and the *k*th occasion, TVP is the typical value of the PK parameter, and *η*_*j*_ and *τ*_*k*_ are the independent and normally distributed between- and within-patient random variability with mean of zero and variance Ω_*P*_ and Π_*P*_, respectively. Different combinations of η correlation (ω-block) and η fixed at zero were evaluated. The selection of an ω-block, if any, was made on the basis of the decrease of the objective function value (OFV). The residual variability was evaluated using a proportional error model according to Eq. (2):

2 $$ C_{\text{obs}} = C_{\text{pred}} \cdot \left( { 1+ \varepsilon } \right) $$

where *C*_obs_ was the observed plasma cabazitaxel concentration; *C*_pred_ was the corresponding model predicted concentration; and ε was the departure of the observed from the predicted concentration, which was assumed to follow a random normal distribution with a mean of 0 and variance, Σ.

#### Covariate analyses

The relationship between individual estimates and covariates was initially investigated graphically. Demographic and disease characteristics including age, sex, height, weight, BSA, race, renal function (creatinine CL), hepatic function (alanine aminotransferase [ALT], aspartate aminotransferase [AST], alkaline phosphatase [ALP], and bilirubin), disease status (tumor type), and concomitant medication with CYP inducers (e.g., prednisone/prednisolone) were tested as potential model covariates. Each covariate was also tested independently using NONMEM^®^.

Since the individual laboratories that analyzed study samples defined their own upper limit of normal (ULN), laboratory values were normalized to their respective ULN prior to evaluation. All continuous covariates were centered to the median value prior to analysis. If any covariates were completely missing for a particular patient and if at least 5 % of the population was also missing this covariate, a number of options were available. If the covariate was categorical, the patient was deleted from the covariate analysis. If the covariate was continuous, the missing value was imputed using the population median of the covariate or, if a strong correlation existed between any other covariates (e.g., weight and BSA), the linear regression was used to calculate the missing covariate value.

Each covariate was added individually to the model (forward selection) using a linear or power model. Continuous covariates were incorporated as shown in Eqs. (3–6):

3 $$ {\text{TVP}} = \theta_{ 1} + \theta_{ 4} \cdot \left( {{\text{COV}}/{\text{Median COV}}} \right) $$

4 $$ {\text{TVP}} = \, \theta_{ 1} + \theta_{ 4} \cdot \left( {{\text{COV}}-{\text{Median COV}}} \right) $$

5 $$ {\text{TVP}} = \theta_{ 1} \cdot \left( {{\text{COV}}/{\text{Median COV}}} \right)**\;\theta_{ 4} $$

6 $$ {\text{TVP}} = \theta_{ 1} \cdot {\text{COV}}/\left( {\theta_{ 4} + {\text{COV}}} \right) $$

where TVP is the typical value (population parameter) of either CL or V1, and COV is the considered continuous covariate.

Discrete covariates were incorporated as shown in Eqs. (7) & (8):

7 $$ {\text{TVP}} = \theta_{ 1} \cdot \left( { 1- \theta_{ 4} \cdot {\text{CAT}}} \right) $$

8 $$ {\text{TVP}} = \theta_{ 1} \cdot {\text{CAT}}**\;\theta_{ 4} $$

where CAT is the considered categorical covariate.

Statistical significance was indicated by a *p* value of ≤0.05. Only covariates providing a significant change in the OFV were included in the full model and were then tested in a backward deletion step, with statistical significance indicated by a *p* value of ≤0.001. The population parameters were re-estimated in consideration of their relationship with the covariates. Finally, the individual PK parameter estimates and some derived exposure variables were calculated using the final model.

#### Model qualification

Parameter precision and model stability were estimated for the final model by a non-parametric bootstrap procedure. A total of 1,000 replicate datasets were created from the original dataset by sampling with replacement using the individual patient as the sampling unit. Each of these datasets was fit to the final model using NONMEM. The parameter estimates from the successful fits were then collected, and empirical 95 % confidence intervals were constructed by computing the 2.5th and 97.5th percentiles for each parameter.

The predictivity of the model was evaluated with a visual predictive check [16]. Cabazitaxel concentrations were simulated for 1,000 patients receiving one 25 mg/m^2^ dose every 3 weeks for three cycles. The infusion duration, BSA, and tumor type were resampled from the total dataset with replacement. The parameter estimates from the final model were used for simulation of the concentrations in NONMEM^**®**^. The non-parametric 90 % confidence interval around the median was computed for each time point and visually compared with the observed dose-normalized concentrations.

## Results

A total of 170 patients (60 women; 110 men) with 4–50 sampling points from 1 to 3 cycles per patient were included in the total dataset. A summary of patient characteristics with relevance to the population PK analysis is presented in Table 3.

**Table 3:** Summary of patient characteristics (*N* = 170)

Parameter	TED6188	TED6189	TED6190	ARD6191	EFC6193	All studies
Age, years^a^	53.3 (18.6) [55.0, 34.0–67.0]	50.9 (25.2) [54.0, 31.0–70.0]	58.9 (18.9) [59.0, 25.0–80.0]	55.1 (21.9) [56.0, 28.0–77.0]	68.0 (10.5) [69.0, 52.0–83.0]	60.4 (19.5) [63.0, 25.0–83.0]
Height, cm^a^	165 (4.78) [168,147–180]	164 (6.96) [166, 144–184]	172 (4.85) [173, 152–185]	159 (4.61) [160, 143–178]	173 (4.20) [174, 155–187]	168 (5.69) [169, 143–187]
Weight, kg^a^	65.3 (17.9) [63.0. 42.0–84.0]	68.1 (21.5) [69.0, 39.0–94.0]	80.6 (22.6) [77.6, 50.1–133]	67.8 (20.9) [68.0, 35.0–105]	80.8 (17.1) [80.0, 54.0–119]	75.3 (21.3) [74.8, 35.0–133]
BSA, m^2a^	1.72 (10.2) [1.69, 1.31–2.04]	1.74 (12.9) [1.80, 1.31–2.17]	1.93 (11.7) [1.89, 1.53–2.53]	1.69 (9.99) [1.71, 1.30–2.07]	1.94 (9.08) [1.97, 1.55–2.37]	1.85 (11.9) [1.84, 1.30–2.53]
BMI, kg/m^2a^	23.9 (14.9) [23.7, 19.1–32.2]	25.3 (19.1) [23.8, 16.7–32.9]	27.2 (19.0) [27.2, 18.6–39.0]	26.8 (21.7) [26.8, 13.3–40.5]	27.0 (15.1) [26.9, 18.4–37.9]	26.5 (18.1) [26.2, 13.3–40.5]
Male, *n* (%)	12 (57.1)	5 (38.5)	26 (74.3)	0	67 (100)	110 (64.7)
Female, *n* (%)	9 (42.9)	8 (61.5)	9 (25.7)	34 (100)	0	60 (35.5)
Race, *n* (%)						
Caucasian	21 (100)	13 (100)	25 (71.4)	31 (91.2)	54 (80.6)	144 (84.7)
Black	0	0	2 (5.7)	1 (2.9)	1 (1.5)	4 (2.4)
Oriental	0	0	1 (2.9)	0	8 (11.9)	9 (5.3)
Hispanic	0	0	7 (20.0)	0	0	7 (4.1)
Other	0	0	0	2 (5.9)	4 (6.0)	6 (3.5)
Tumor type, *n* (%)						
Prostate	0	0	10 (28.6)	0	67 (100)	77 (45.3)
Breast	0	3 (23.1)	0	34 (100)	0	37 (21.8)
Gastrointestinal	6 (28.6)	6 (46.2)	11 (31.4)	0	0	23 (13.5)
Other	15 (71.4)	4 (30.8)	14 (40)	0	0	33 (19.4)
Renal function						
CrCl, mL/min^b^	91.7	77.9	84.7	95.7	89.6	89.2
Liver function^c^						
ALT ratio	0.322	0.891	0.537	0.755	0.605	0.608
AST ratio	0.471	0.940	0.869	0.881	0.941	0.856
ALP ratio	0.765	1.35	1.52	0.968	2.20	1.57
Bilirubin ratio	0.582	0.479	0.377	0.440	0.438	0.447
Concomitant CYP inducer, *n* (%)^d^						
First cycle	0	0	0	0	0	0
Other cycles	0	N/A	0	N/A	48/51 (94.1)	48/100 (48)

*CrCl* Creatinine clearance, *CV* coefficient of variation, *BSA* body surface area, *ALT* alanine aminotransferase, *AST* aspartate aminotransferase, *ALP* alkaline phosphatase, *CYP* cytochrome P450

^a^Results presented as mean (CV %) [median, min–max]

^b^Among the 170 patients, 59 patients had mild renal impairment (50 mL/min ≤ CrCl ≤ 80 mL/min), 14 patients had moderate renal impairment (30 mL/min ≤ CrCl < 50 mL/min), and only 1 patient had severe renal impairment (CrCl < 30 mL/min)

^c^Since the individual laboratories that analyzed study samples defined their own upper limit of normal, values are displayed as a normalized ratio of the upper limit of normal

^d^Mainly prednisone/prednisolone; 32 patients received prednisone or prednisolone at cycle 2

Prior to model selection, one obvious outlier concentration was removed and statistical outlier detection led to the removal of a further 66 samples (2.8 % of the number of concentrations in the initial dataset). This did not lead to the loss of any patient. Of note, a total of 322 samples were below the LOQ. However, 151 of these were taken prior to drug administration in cycle 1, and 94 were taken prior to drug administration in cycle 2 or cycle 3; in total, 245 LOQ samples (76 %) were taken prior to drug administration. Concentrations below the LOQ were excluded from the analysis. The total dataset was composed of 170 patients with 2,322 measurable concentrations.

Two- and three-compartment models were evaluated with CL and volume parameterization (ADVAN11, TRANS4) or with micro rate constant parameterization (ADVAN11, TRANS1). A three-compartment model was found to better describe the data than a two-compartment model, with a decrease in the OFV of 930. The best base model (Online Resource 1) was a three-compartment open model with first-order elimination (CL) from the central compartment (V1) and intercompartmental rate constant parameterization (ADVAN11, TRANS1), IIV on all parameters except K21 and a proportional residual error. Addition of IOV in CL and V1 significantly improved the OFV (decrease in OFV of 247). The stability of the model was evaluated over 25 runs, in which initial parameter estimates were modified using estimates obtained from the base model and a coefficient of variation of 50 % for the distribution of alternative starting points. Twenty-two runs were successful, and descriptive statistics computed from these runs confirmed model stability.

Before screening for covariate effect on CL and V1, the correlation between covariates was evaluated graphically and by calculating the correlation coefficients. No major relationships were observed between covariates, except for the self-evident associations between female sex and breast cancer, and of height and weight with BSA.

The relationships between population parameters and covariates were identified graphically by plotting the η values for CL and V1 against the covariates. These plots revealed links between CL and sex, BSA, AST ratio, ALT ratio, and tumor type. They also revealed a link between V1 and sex, BSA (plus height and weight), and renal function (creatinine CL). A univariate population PK screening was then performed to confirm that these covariates were statistically significant, having met the criteria for consideration in the model-building step. In step 1 of covariate selection, BSA, height, weight, sex, breast cancer tumor type, ALT ratio, and inducers were all significant covariates for CL, and BSA, weight, Caucasian race, and gastrointestinal tumor type were all significant covariates for V1. Due to the clear relationship and high degree of correlation between BSA, height, and weight, only BSA (which led to the most significant decrease in OFV) was incorporated into the full model. The power term initially evaluated for BSA effect was not retained in the model as its inclusion resulted in a non-significant 2.1-point change in OFV. The effect of ALT ratio on CL was not included in the full model as the 95 % confidence interval of its covariate effect included zero.

The final population PK model obtained from the total dataset allowed identification of a significant relationship between BSA, breast cancer tumor type, and CL as shown in Eq. (9):

9 $$ {\text{CL}}(L/h )= 4 8. 5\cdot \frac{\text{BSA}}{1.84} \cdot (1 - {\text{TT}}1 \cdot 0.543) $$

where 1.84 m^2^ was the median BSA value and TT1 is 1 for breast cancer and 0 for other cancers. The inclusion of these two covariates led to a modest, but statistically significant, reduction in OFV of 32.0 and in IIV in CL from 47.7 to 38.8 % from the base model. Sex, weight, height, BMI, age, Caucasian race, renal and hepatic function, and concomitant use of CYP inducers were not retained as covariates. Final population PK parameters are presented in Table 4. With the final model, the mean CL, V1, and *V*_ss_ in non-breast cancer patients (assumed BSA: 1.84 m^2^) were 48.5 L/h (26.4 L/h/m^2^), 26.0 L (14.1 L/m^2^), and 4,870 L (2,640 L/m^2^), respectively. The *t*_1/2_s for α, β, and γ phases were 4.4 min, 1.6, and 95 h, respectively.

**Table 4:** Cabazitaxel population PK model (*N* = 170)

	NONMEM					Bootstrap			
Parameter	Estimate	RSE, %	95 % CI (lower)	95 % CI (upper)	Shrinkage %	Estimate	RSE, %	95 % CI (lower)*	95 % CI (upper)*
$\theta_{ 1} {\text{in}}\,\left[ {{\text{CL}} = \theta_{ 1} \cdot {\text{BSA}}/ 1. 8 4\cdot \left( { 1- \theta_{ 7} \cdot {\text{TT1}}} \right)} \right],{\text{ L}}/{\text{h}}$	48.5	5.48	43.2	53.8	N/A	48.6	5.35	43.5	53.7
*θ*_2_ (V1, L)	26.0	12.5	19.5	32.5	N/A	26.0	12.0	19.9	32.1
*θ*_3_ (K12, h^−1^)	2.48	9.60	2.00	2.95	N/A	2.46	10.7	1.95	2.97
*θ*_4_ (K21, h^−1^)	0.604	6.67	0.524	0.685	N/A	0.611	6.76	0.530	0.692
*θ*_5_ (K13, h^−1^)	4.84	10.4	3.83	5.85	N/A	4.90	9.67	3.97	5.83
*θ*_6_ (K31, h^−1^)	0.0266	5.94	0.0234	0.0297	N/A	0.0267	6.55	0.0233	0.0301
$\theta_{ 7} {\text{in}}\,\left[ {{\text{CL}} = \theta_{ 1} \cdot {\text{BSA}}/ 1. 8 4\cdot \left( { 1- \theta_{ 7} \cdot {\text{TT1}}} \right)} \right]$	0.543	30.0	0.217	0.869	N/A	0.536	42.2	0.0930	0.979
Interindividual variability (CV, %)									
CL	38.8	32.6	22.8	49.8	30.4	34.5	20.3	20.7	48.3
V1	93.4	16.9	76.0	108.0	14.0	93.7	8.67	77.8	110
K12	84.0	31.3	51.4	107.0	14.1	84.1	12.8	62.9	105
K13	64.2	22.0	48.0	77.0	12.8	63.5	12.3	48.3	78.7
K31	28.2	28.7	18.4	35.4	35.4	28.4	14.4	20.4	36.4
Interoccasion variability (CV, %)									
CL	19.4	41.0	8.22	26.2	58.0	20.0	22.6	11.1	28.9
V1	45.3	33.2	26.2	58.4	42.9	42.4	19.5	26.2	58.6
Secondary parameters									
*V*_ss_, *L*	4,870	N/A	3,290	6,570	N/A	N/A	N/A	N/A	N/A
*t*_1/2α_, min	4.44	N/A	5.08	3.90	N/A	N/A	N/A	N/A	N/A
*t*_1/2β_, h	1.58	N/A	1.77	1.41	N/A	N/A	N/A	N/A	N/A
*t*_1/2λ_, h	95.1	N/A	81.8	108	N/A	N/A	N/A	N/A	N/A
Residual variability (CV, %)									
σ (proportional)	27.8	6.71	25.9	29.6	N/A	28.1	7.38	25.9	30.0

*RSE* Percentage of relative standard error (100 % * SE/estimate), expressed as percentage (%), *CI* confidence interval, *θ* population PK parameters, *N*/*A* not applicable, *BSA* body surface area, *CV* coefficient of variation, *CL* clearance, *V*1 central volume of distribution, *K*12, *K*21, *K*13, *K*31 intercompartmental rate constants, *L* liter, *TT*1 breast cancer tumor type, *σ* associated variance of the residual error variable (ε)

* Empirical 95 % confidence intervals were constructed by computing the 2.5th and 97.5th percentiles for each parameter

**Fig. 1:**
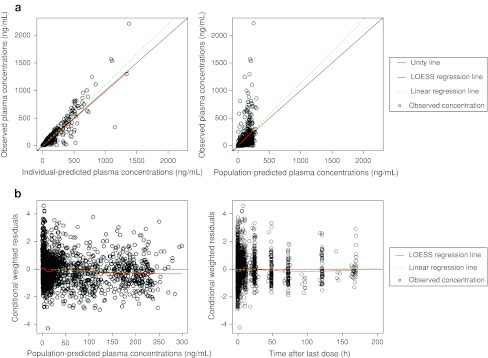
**a** Relationship between population-predicted or individual-predicted and observed plasma concentrations **b** relationship between population conditional-weighted residuals and population-predicted concentrations or time from last dose.* Circle*: observed concentration;* black line*: unity line;* red line*: Loess regression line;* green dashed line*: linear regression line

Selected goodness-of-fit plots showing the adequacy of the model are presented in Fig. 1.

CL values for the 5th (1.50 m^2^) and 95th (2.22 m^2^) percentile of BSA were 39.5 L/h and 58.5 L/h, respectively (percentage change from CL [48.5 L/h] for median BSA of 1.84 m^2^: −18.5 and 20.7 %, respectively). Full concentration–time profiles for a 25 mg/m^2^ dose of cabazitaxel are shown in Online Resource 2.

### Model verification

**Fig. 2:**
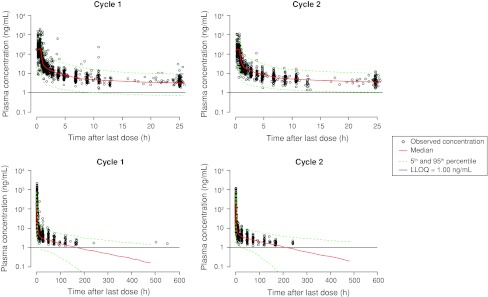
Visual predictive check of plasma concentration versus time normalized to a therapeutic dose of 25 mg/m^2^ for cycle 1 (*left*) and cycle 2 (*right*) from 0 to 25 h (*top*) and 0 to 600 h (*bottom*) from previous dose.* Circle* observed concentration;* red line* median;* green dotted line* 5th and 95th percentile; LLOQ: lower limit of quantification

Bootstrapping was performed as described previously. From the original total dataset of 170 patients, 1,000 runs were launched, of which 712 were successful. The majority of the failed runs (54 %) were caused by rounding errors with significant digits less than or equal to 3, with the other main reason being convergence of the objective function. The condition number (ratio of highest to lowest eigenvalue) was 56. For each successful run, population PK parameters and corresponding mean, standard deviation, and 2.5th and 97.5th percentiles were computed. These values are presented in Table 4 and are very similar to, if not the same as, those obtained with the final model using NONMEM^**®**^, confirming the robustness and accuracy of the final parameters. When using all 1,000 bootstrap runs, the summary statistics are virtually identical to the values presented in Table 4. Visual predictive checks were also performed (Fig. 2). These show the adequate predictivity of the model.

### Empirical Bayes estimates and derived exposure variables

Empirical Bayes estimates and α, β, and γ *t*_1/2_s are summarized in Table 5 for all patients and by study.

**Table 5:** Summary statistics of empirical Bayes estimates from the first available cycle by study and cycle (*N* = 170). Results are presented as arithmetic mean (CV, %)

Group	CL, L/h/m^2^	V1, L/m^2^	*V*_ss_, L/m²	*t*_1/2α_, min	*t*_1/2β_, h	*t*_1/2γ_, h
All (*n* = 170)	24.2 (40.2)	19.6 (82.9)	3,360 (66.3)	4.60 (55.6)	1.78 (45.9)	134 (63.3)
TED6188 (*n* = 21)	34.5 (25.2)	17.1 (59.4)	3,660 (51.9)	3.57 (32.4)	1.56 (13.9)	108 (54.5)
TED6189 (*n* = 13)	25.5 (36.4)	16.0 (73.1)	3,160 (66.1)	3.38 (40.7)	1.90 (43.8)	130 (71.5)
TED6190 (*n* = 35)	24.2 (36.2)	10.8 (86.6)	2,710 (84.4)	2.85 (35.7)	1.57 (21.9)	103 (51.1)
ARD6191 (*n* = 34)	12.1 (26.1)	20.5 (74.3)	3,270 (63.5)	4.99 (40.7)	2.39 (62.6)	210 (55.7)
EFC6193 (*n* = 67)	27.0 (24.1)	25.2 (77.5)	3,700 (64.0)	5.88 (52.3)	1.62 (21.0)	120 (49.9)

*CL* Clearance, *V1* central volume of distribution, *V*_*ss*_ volume of distribution at steady state, *t*_*1/2*_ half-life, *α*, *β*, *γ* 1st, 2nd, 3rd phase of decay

## Discussion

Population PK modeling is an important tool for the investigation of PK and PD variability, and dose–concentration–effect relationships, and provides information that can be useful for the registration process of new agents [9]. A population PK model for cabazitaxel was developed and validated using data obtained from 170 patients with advanced solid tumors enrolled in five studies across the agent’s Phase I–III clinical development. The PK of cabazitaxel was best described by a three-compartment model with IIV and IOV on CL and V1, IIV on all intercompartmental rate constants except K21, and with proportional residual error. Sex, BMI, age, Caucasian race, renal and hepatic function, and concomitant use of CYP inducers did not significantly affect the model and were not retained as covariates of cabazitaxel CL or V1 in the analyzed patient dataset. Cabazitaxel PK was not altered in patients with mild (50 mL/min ≤ creatinine CL [CrCl] ≤ 80 mL/min; *n* = 59) and moderate renal impairment (30 mL/min ≤ CrCl < 50 mL/min; *n* = 14), suggesting no dose adjustment is necessary in these populations. These results are expected as cabazitaxel renal elimination is minimal (2.3 % as unchanged drug) [8], as was observed with other taxanes (docetaxel and paclitaxel) [6, 17]. However, patients with severe renal impairment (CrCl < 30 mL/min) and end-stage renal disease should be treated with caution and monitored carefully during treatment as only one patient with severe renal impairment was included in this analysis.

A total of 66 plasma concentrations were excluded as outliers as they were considerably higher than the mean plasma concentration at the corresponding time points. Most were obtained during or close to IV infusion. It is likely that these outliers were related to improper but non-documented samplings (for example, sampling at the injection site). To assess the effect of exclusion of the 66 outlier concentrations, a re-analysis was conducted in which these concentrations were included. CL estimates were 31 % lower than in the original analysis, which was as expected based on the high concentrations in the excluded samples.

The IIV of cabazitaxel CL was significantly related to BSA and breast cancer tumor type. The effect of BSA on CL is addressed in the clinic by BSA-dependent dosing. The validity of this approach is shown in Online Resource 3, in which η_CL_ is plotted against BSA both before and after inclusion of covariates in the final pharmacostatistical model. These data demonstrate graphically that dosing by BSA is appropriate. Conversely, the observed effect of breast cancer in reducing CL by 54 % compared with other types of tumor is unexpected. There was a large uncertainty around this effect, as shown by the wide 95 % confidence interval of 9.3 to 97.9 % reduction obtained with the non-parametric bootstrap. It is possible that the breast cancer effect is a study effect, given that the vast majority of breast cancer patients evaluated (34 of 37) were from a single Phase II study. CL did not appear to be influenced by sex: The 34 patients with breast cancer (drawn from the aforementioned Phase II study) only formed approximately half of the female population (34 of 60) in this pooled analysis. Therefore, any bias from the breast cancer study would have been diluted by the additional female patients from other studies. Indeed, the mean CL value appeared comparable between males (mean: 27.6 L/h/m²; coefficient of variation [CV]: 29 %) and females (mean: 27.0 L/h/m²; CV: 36 %) when removing the 37 patients with breast cancer. Given that the breast cancer finding is highly confounded by study, its clinical relevance is difficult to interpret from the dataset described here. Nevertheless, when the final model was run with sex or study ARD6191 instead of breast cancer tumor type as covariate on CL, the lowest OFV and the strongest covariate effect were obtained when the tumor-type effect on CL was replaced by a study effect (as shown in Online Resource 4). In addition, further information is available from a study in which the PK of cabazitaxel was evaluated in patients with metastatic breast cancer who were treated with cabazitaxel in combination with capecitabine. In that study, the cabazitaxel CL value (33.6 L/h/m²) [18] was comparable to that obtained with cabazitaxel monotherapy in patients with advanced solid tumors (CL 24.2–34.5 L/h/m²), but was much higher than the value predicted in patients with metastatic breast cancer from the study ARD6191 (12.1 L/h/m²) (Table 5). Because capecitabine is not known to induce or inhibit CYP3A [19], and capecitabine did not appear to alter the PK of cabazitaxel [18], the lower plasma CL value observed in study ARD6191 is most likely attributed to a study effect rather than a tumor-type effect. These findings support a study effect on CL rather than a tumor-type effect, with the lowest CL values obtained in patients from the ARD6191 study compared with those estimated in patients from other studies.

The remaining IIV (not explained by covariate effects) was 38.8 % for CL and 93.4 % for V1. Both IOV (19.4 % for CL and 45.3 % for V1) and residual variability (27.8 %) were moderate. In the Phase I studies, plasma samples were collected up to 48–240 h after the end of the infusion, depending on the dose and the study. The population PK analysis allowed a better estimation of the PK parameters from the studies by reducing the impact of sample times. Hence, longer terminal *t*_1/2_, larger *V*_ss_, and lower CL values were estimated by the population PK analysis compared with those obtained by individual modeling (Table 5) [1, 5]. In addition, the population analysis made possible the estimation of the plasma CL, the *V*_ss_, and the *t*_1/2_s (*t*_1/2α_, *t*_1/2β_, and *t*_1/2γ_) in TED6189.

The PK of other taxane agents has been extensively evaluated previously. Twenty-four studies of docetaxel PK found that the three main covariates that predicted CL were BSA, plasma levels of alpha-1-acid glycoprotein (AAG), and hepatic function [12, 20]. However, only hepatic function is likely to have clinical relevance; patients with concomitant elevations of both aminotransferases and ALP demonstrated a 27 % reduction in CL, which was predicted to result in a 1.5-fold increase in the risk of febrile neutropenia [12]. Additional covariates that showed minor but significant effects on docetaxel CL were albumin level and age. These results suggest the presence of slight differences in disposition between docetaxel and cabazitaxel. Cabazitaxel is eliminated in feces mainly as metabolites (following metabolism by the liver) [8]. In this study, although the results of the univariate population PK analysis demonstrated relationships between cabazitaxel CL and liver function tests (i.e., lower CL values with increasing ALT), unlike docetaxel, transaminase levels were found to have no significant impact on cabazitaxel PK (removal of ALT from the full model did not lead to an increase in ΔOFV of greater than 10.8). However, in this study, the number of patients with elevated transaminase findings was small (one patient with a bilirubin ratio above the ULN; 4 and 19 patients with ALT and AST ratios 1.5-fold higher than the ULN, respectively; 18 patients with ALP ratio 2.5-fold higher than the ULN). Therefore, this result should be interpreted with caution. In population PK analysis for docetaxel, AAG was shown to influence variability in CL [12, 20]. Although the effect of AAG was not assessed in the current study, this may be a further source of differences in the distribution of cabazitaxel and docetaxel; while cabazitaxel is highly bound to most plasma proteins, particularly albumin, it is poorly bound to AAG, unlike docetaxel. In addition to the differences in *t*_1/2_ and *V*_ss_ mentioned previously, these results suggest further differences in the distribution and elimination of docetaxel and cabazitaxel, which possibly reflect the differences in their molecular structure.

The PK profile of paclitaxel is complicated by the requirement for co-administration with Cremophor EL (CrEL), which results in the appearance of non-linear PK associated with entrapment within CrEL micelles [21]. However, while the plasma-bound fraction of paclitaxel increases in proportion to the CrEL dose, the higher plasma concentrations do not result in higher concentrations in body tissues [21]. A mechanism-based population PK model suggests that unbound paclitaxel concentration can be predicted from total plasma concentration, providing that the CrEL concentration is known. If not available, covariates including BSA and bilirubin levels can be used, along with dose, to predict plasma concentrations [22]. Compared with cabazitaxel, the IIV of paclitaxel is somewhat lower (19–25 %), while IOV is similar (20 %) [22, 23].

In conclusion, the population PK data described here suggest that cabazitaxel exposure is consistent across most solid tumor types and is not complicated by patient demographics or baseline factors, other than BSA. Although the presence of breast cancer was found to have an effect on cabazitaxel CL, the data contributing to this finding were almost entirely derived from a single study; the finding therefore merits further evaluation. Furthermore, the finding that BSA influences CL is unlikely to be clinically relevant, given that cabazitaxel dose is already adjusted according to this parameter in clinical practice. The PK of cabazitaxel may differ from those of docetaxel, indicating that the two agents have distinct properties. This is supported by the Phase III TROPIC trial [4], in which cabazitaxel was associated with prolonged survival in patients with mCRPC whose disease had progressed during or after treatment with a docetaxel-containing regimen.

Based on the results of the TROPIC trial [4], cabazitaxel (25 mg/m^2^ dose every 3 weeks) was approved by the US Food and Drug Administration, the Canadian Health Authority, and the European Medicines Agency for the treatment of mCRPC that has progressed following docetaxel treatment. The finding that cabazitaxel PK remains predictable across most solid tumor types may be valuable to its further clinical development.

## Electronic supplementary material

Below is the link to the electronic supplementary material.

Supplementary material 1 (DOCX 340 kb)
