# Supplementary material for: Population pharmacokinetics of cabazitaxel in patients with advanced solid tumors
Source: Cancer Chemother Pharmacol. 2013 Jan 9;71(3):681–92. doi: 10.1007/s00280-012-2058-9 (PMC3579428; doi:10.1007/s00280-012-2058-9)
Supplement: Supplementary file 1 — Supplementary material 1 (DOCX 340 kb) [file 280_2012_2058_MOESM1_ESM.docx]

### Online Resource 1

Graphical representation of the pharmacostatistical model

CL: clearance


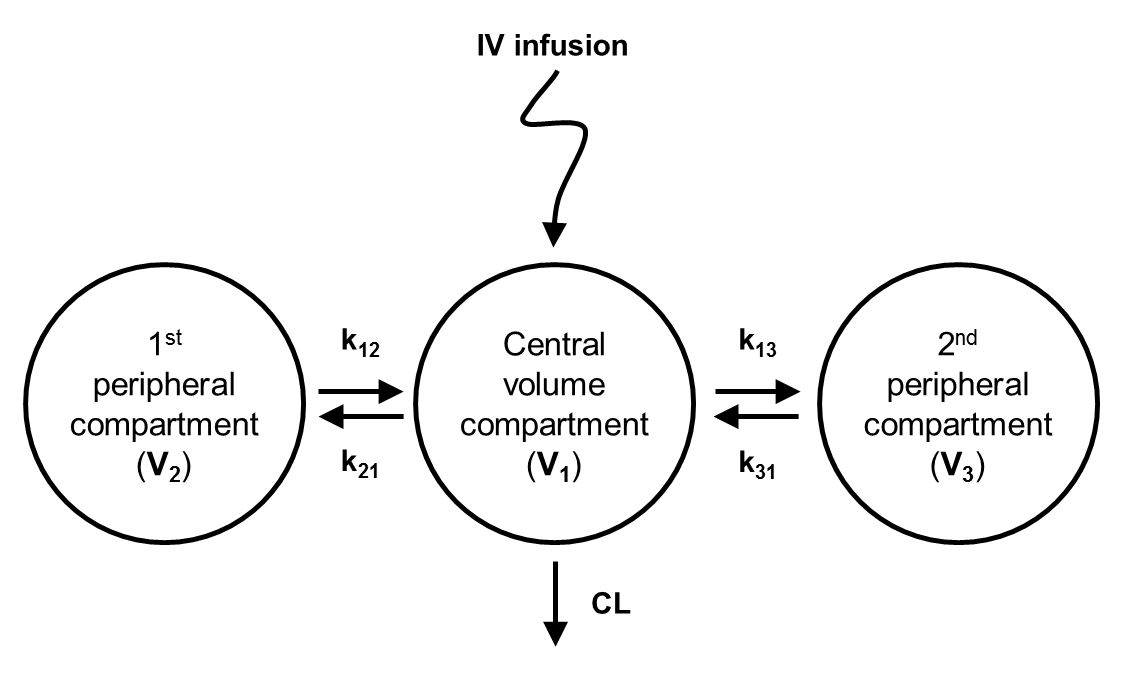


### Online Resource 2

Concentration–time profile for a typical non-breast cancer patient with varying BSA receiving a 25 mg/m^2^ dose (Cartesian scale, 0–4 h, left; logarithmic scale, 0–240 h, right)

Solid line: BSA of 1.84 m^2^ (median BSA); dashed line: BSA of 1.50 m^2^ (5^th^ percentile BSA); dotted line: BSA of 2.22 m^2^ (95^th^ percentile BSA); BSA: body surface area

###
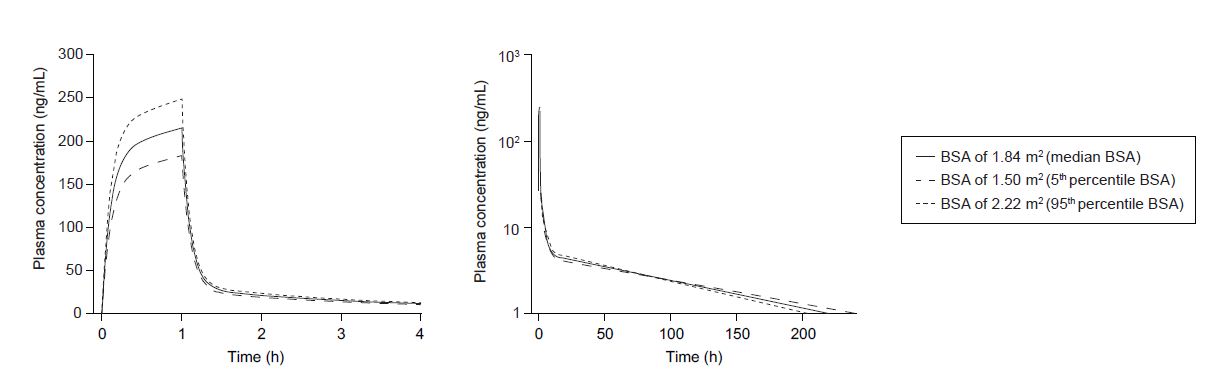


### Online Resource 3

Relationship between η_CL_ and body surface area in the total dataset

Red line: LOESS regression line; green line: linear regression line

1. Before inclusion of covariates (pharmacostatistical model)

1. After inclusion of covariates (final model)

### BSA: body surface area; ETCL: η_CL_ Online Resource 4

Evaluation of effect of sex and study ARD6191 on final model, and comparison with effect of breast cancer

| **Model run** | **OFV** | **Covariate effect (95% CI)** |
| --- | --- | --- |
| Final model with breast cancer on CL | 10137 | 0.543 (0.217–0.869) |
| Final model with sex replacing breast cancer on CL | 10148 | 0.266 (0.0230–0.0309) |
| Final model with study ARD6191 replacing breast cancer on CL | 10131 | 0.637 (0.268–1.01) |

CI: confidence interval; CL: clearance; OFV: objective function value
